# A multi-sensor human gait dataset captured through an optical system and inertial measurement units

**DOI:** 10.1038/s41597-022-01638-2

**Published:** 2022-09-07

**Authors:** Geise Santos, Marcelo Wanderley, Tiago Tavares, Anderson Rocha

**Affiliations:** 1 University of Campinas, Institute of Computing, Campinas, Brazil; 2 McGill, Music Tech, Montreal, Canada; 3 University of Campinas, School of Electrical and Computer Engineering, Campinas, Brazil

**Keywords:** Computer science, Scientific data

## Abstract

Different technologies can acquire data for gait analysis, such as optical systems and inertial measurement units (IMUs). Each technology has its drawbacks and advantages, fitting best to particular applications. The presented multi-sensor human gait dataset comprises synchronized inertial and optical motion data from 25 participants free of lower-limb injuries, aged between 18 and 47 years. A smartphone and a custom micro-controlled device with an IMU were attached to one of the participant’s legs to capture accelerometer and gyroscope data, and 42 reflexive markers were taped over the whole body to record three-dimensional trajectories. The trajectories and inertial measurements were simultaneously recorded and synchronized. Participants were instructed to walk on a straight-level walkway at their normal pace. Ten trials for each participant were recorded and pre-processed in each of two sessions, performed on different days. This dataset supports the comparison of gait parameters and properties of inertial and optical capture systems, whereas allows the study of gait characteristics specific for each system.

## Abstract (summary)

**Table:** 

Measurement(s)	G Force • Spatial Orientation
Technology Type(s)	Accelerometer • Optical Instrument

## Background & Summary

Gait analysis has been explored since the 17th century^1^. Advances in understanding the human motion throughout the last centuries allowed researchers apply this analysis to many applications, such as clinical assessment, monitoring of sports and athletic performances, rehabilitation support, robotics research, and biometry-based recognition^2^. This type of analysis can use data acquired by means of different technologies, like optical systems, inertial measurement units (IMUs), force-plate platforms, force shoes, and techniques based on computer vision. Although some of these are commonly used in most fields, as optical systems and imaging techniques, each technology has its drawbacks and fits best to particular applications^3–7^.

One popular technology to acquire data for gait analysis is the optical motion capture system. It has minimal impact on the natural motion of the participant, as it does not need tethering any hardware onto the individual^8^. This system also fosters a precise acquisition of physical movements over virtual modeling and accurate reconstruction of movement marks and participants’ geometry^9^. However, optical motion capture systems are usually expensive, require high-speed processing devices and specific installations in a controlled space for their use^3^.

Recently, IMUs have been considered an appropriate option to perform gait analysis because they mitigate these drawbacks of optical motion capture systems. They are typically more cost-effective, do not need a controlled environment, and support indoor and outdoor places. However, they have other limitations as being more susceptible to drift caused by changes in motion direction, and to low-frequency noise from small vibrations during the capture. Also, the sensor attachment position significantly impacts the estimation of gait parameters^3,9–11^.

Several important gait datasets comprising either optical motion capture data or inertial data have been made available^12–18^ in the prior literature. However, there are currently no datasets with data being simultaneous captured from both systems, which may allow a multi-modal gait analysis. This synchronized capture has proven helpful in specific applications, such as music gesture analysis^19^ and sports science^20^. We propose, in this work, a multi-sensor gait dataset, which consists of inertial and optical motion data, and aims to provide basis for comparison and reasoning of human gait analysis using data from both systems.

The presented dataset comprises inertial and optical motion data from 25 participants free of lower-limb injuries, aged between 18 and 47 years. A smartphone and a custom microcontroller device with an IMU were attached to one of the participant’s legs to capture accelerometer and gyroscope data, and 42 reflexive markers were taped over the whole body to record three-dimensional trajectories. The participants were instructed to walk on a straight-level walkway at their normal pace. The custom device uses a wireless protocol to communicate with the computer to which the optical system was connected. This setup enabled recording and synchronizing the trajectories (acquired by the optical system) and the inertial measurements (acquired by the dedicated device and the smartphone). Ten trials for each participant were recorded and pre-processed in each of two sessions, performed on different days. This amounts to 500 trials of three-dimensional trajectories, 500 trials of accelerometer and gyroscope readings from the custom device, and 500 trials of accelerations from the smartphone.

In addition to contributing with a multi-sensor dataset which supports the comparison of gait parameters and properties of inertial and optical capture systems, the full-body marker set and the inertial sensors attached to the leg favor the study of gait characteristics specific for each system. This dataset also allows analyzing gait variations between participants and for each one (i.e., intra and inter-participants) by the captures in different days. This characteristic of the dataset fosters investigations about the effectiveness of gait recognition and user profiling using inertial data.

## Methods

### Participants

Twenty-five participants (12 women, 12 men, and one undeclared gender, aged between 18 and 45 years) participated in this study, which took place between December of 2019 and February of 2020. Neither of them reported injuries for both legs or medical conditions that would affect their gait or posture. The participants were either students of the School of Music at McGill University, members of CIRMMT, or authors of this work. McGill University’s Research Ethics Board Office approved this study (REB File # 198–1019), and all participants provided informed consent.

### Experimental design

**Fig. 1:**
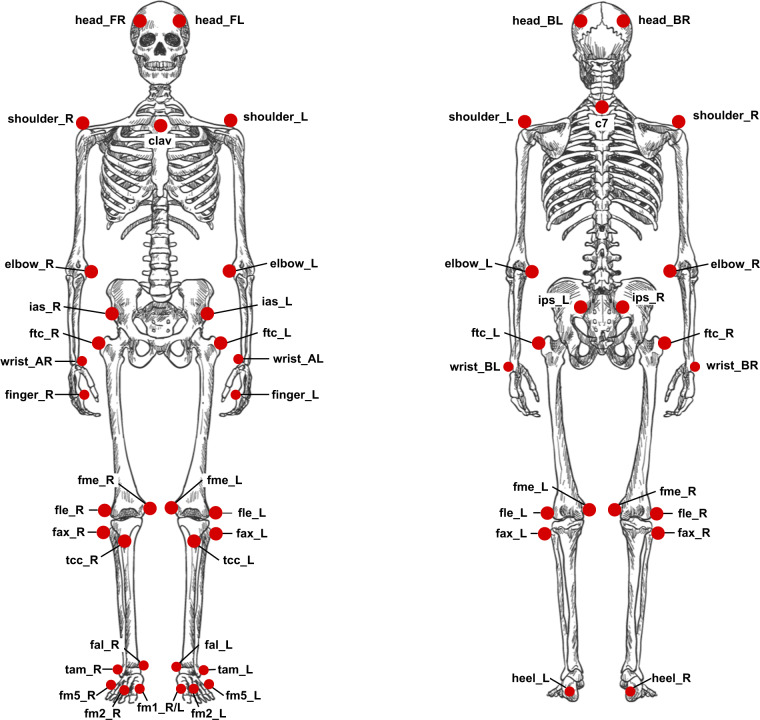
The adopted marker set based in the model proposed by Dumas and Wojtusch and the Plug-in Gait model.

A proprietary optical system^21^, with 18 infra-red Oqus 400 and Oqus 700 cameras and sampled at 100 Hz, was adopted to track the 3D trajectories of 42 reflective markers over the participants’ body. The marker set was based on the lower-limb IORGait model proposed by Leardini *et al*.^22^, and a simplified upper-limb and trunk Plug-in Gait models^23^. This marker set is depicted in Fig. 1, and each reflexive marker, as well as its anatomical landmarks, are described in Table 1. Gait analysis usually focuses on the lower limb trajectories; thus, the upper-limb and trunk simplified models are used only for skeleton reconstruction purposes. The marker placement was performed by anatomical palpation using the landmarks reported in Table 1 at the beginning of each session, and was not changed during the session trials. The proprietary motion capture software (*Qualisys Track Manager* - QTM)^24^ was employed to record and pre-process the 3D trajectories of the reflexive markers. All trajectory measurements were acquired in units of millimeters.

**Table 1:** Description and placement of each reflexive marker attached to the participants’ body.

Labels	Description	Landmarks for marker placement
top_right	Marker on the top right of the band	Located approximately on the top right of the smartphone
top_left	Marker on the top left of the band	Located approximately on the top left of the smartphone
top_imu	Marker on the center of the band	Located approximately on the top of the MCU’s box
head_imu	Marker on the left half of the band	Located approximately on the left side of the MCU’s box
power	Marker on the right half of the band	Located approximately on top of the powerbank used as power source to the MCU
finger	Index finger	Placed on the distal phalanx of the right/left index finger
head_FR	Right front head	Located approximately over the right temple
head_FL	Left front head	Located approximately over the left temple
head_BR	Right back head	Placed on the back of the head, roughly in a horizontal line of the right front head marker
head_BL	Left left head	Placed on the back of the head, roughly in a horizontal line of the left front head marker
shoulder_R	Right shoulder	Placed on the acromio-clavicular joint
shoulder_L	Left shoulder	Placed on the acromio-clavicular joint
c7	7th Cervical Vertebrae	Spinous process of the 7th cervical vertebrae
clav	Clavicule	Jugular Notch where the clavicles meet the sternum
elbow_R	Right elbow	Lateral epicondyle approximating elbow joint axis
elbow_L	Left elbow	Lateral epicondyle approximating elbow joint axis
wrist_BR	Right wrist marker B	Right wrist bar pinkie side
wrist_AR	Right wrist marker A	Right wrist bar thumb side
wrist_AL	Left wrist marker A	Left wrist bar thumb side
wrist_BL	Left wrist marker B	Left wrist bar pinkie side
finger_R	Right finger	Dorsum of the right hand just below the head of the second metacarpal
finger_L	Left finger	Dorsum of the left hand just below the head of the second metacarpal
ips_R	Left PSIS	Right posterior-superior iliac spine
ips_L	Left PSIS	Left posterior-superior iliac spine
ias_R	Right ASIS	Right anterior-superior iliac spine
ias_L	Left ASIS	Left anterior-superior iliac spine
ftc_R	Right greater trochanter	Most lateral prominence of the right greater trochanter
ftc_L	Left greater trochanter	Most lateral prominence of the left greater trochanter
fme_R	Left medial femoral epicondyle	Most medial prominence of the left medial femoral epicondyle
fme_L	Right medial femoral epicondyle	Most medial prominence of the right medial femoral epicondyle
tcc_R	Right tibial tuberosity	Most anterior border of the right tibial tuberosity
ttc_L	Left tibial tuberosity	Most anterior border of the left tibial tuberosity
fax_R	Right fibula head	Proximal tip of the head of the right fibula
fax_L	Left fibula head	Proximal tip of the head of the left fibula
fle_R	Right lateral femoral epicondyle	Most lateral prominence of the right lateral femoral epicondyle
fle_L	Left lateral femoral epicondyle	Most lateral prominence of the left lateral femoral epicondyle
heel_R	Right heel	Right posterior calcaneus
heel_L	Left heel	Left posterior calcaneus
fal_R	Right lateral malleolus	Lateral prominence of the right lateral tibial malleolus
fal_L	Left lateral malleolus	Lateral prominence of the left lateral tibial malleolus
tam_R	Right medial malleolus	Most medial prominence of the right medial tibial malleolus
tam_L	Left medial malleolus	Most medial prominence of the left medial tibial malleolus
fm5_R	Right 5th metatarsal head	Dorsal margin of the right fifth metatarsal head
fm5_L	Left 5th metatarsal head	Dorsal margin of the left fifth metatarsal head
fm2_R	Right 2nd metatarsal head	Dorsal aspect of the right second metatarsal head
fm2_L	Left 2nd metatarsal head	Dorsal aspect of the left second metatarsal head
fm1_R	Right 1st metatarsal head	Dorsal margin of the right first metatarsal head
fm1_L	Left 1st metatarsal head	Dorsal margin of the left first metatarsal head

A Nexus 5 Android-based smartphone with an InvenSense MPU-6515 six-axis IMU was used to capture accelerometer data. An Android application was designed and developed to read accelerations at a sampling rate of 100 Hz and store them into a comma-separated values (CSV) file. The accelerometer measurements yielded by the Android platform are in units of *m*/*s*^2^.

**Fig. 2:**
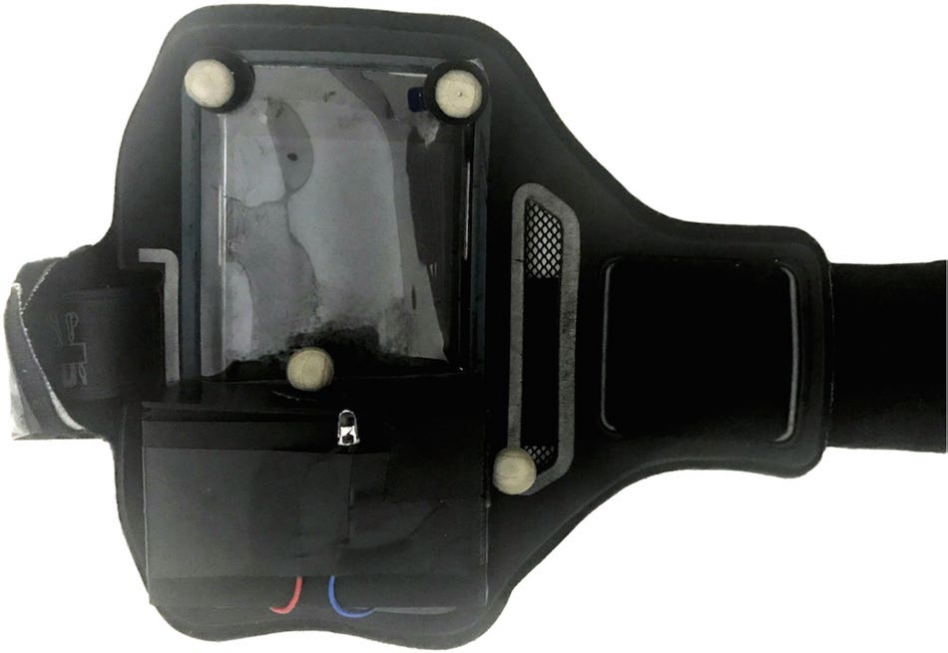
The smartphone and MCU box mounted to a band to be attached to the leg.

**Fig. 3:**
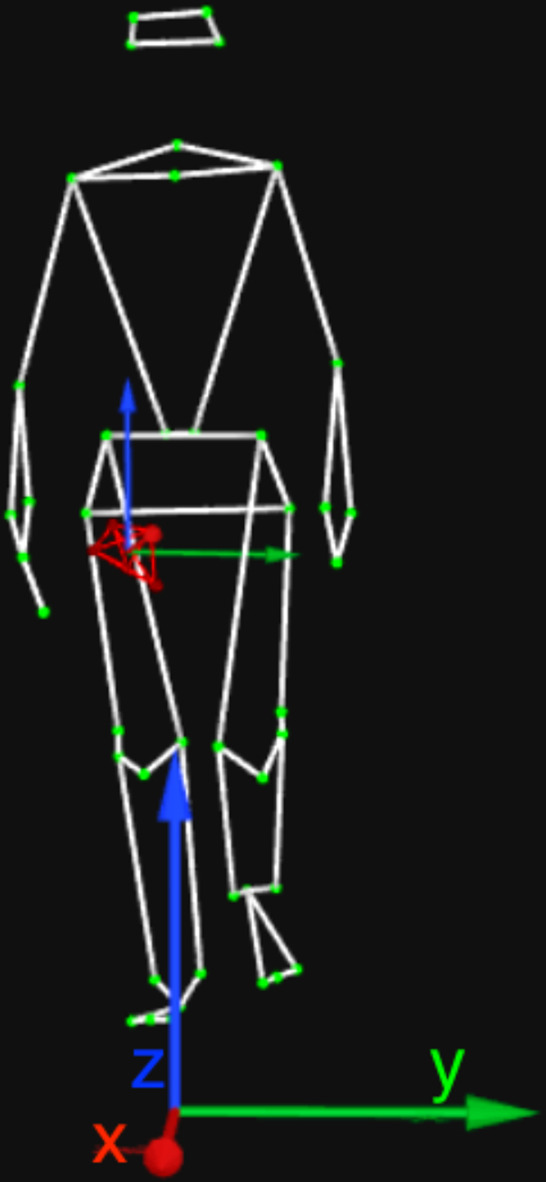
Coordinate reference of the motion capture system, and the smartphone and MCU attached to the leg being tracked as a rigid body.

An InvenSense MPU-9250^25^ six-axis IMU mounted to an ESP8266 microcontroller (MCU) was used to capture accelerometer and gyroscope data. A firmware to the ESP8266 was developed using Arduino Core libraries, to read raw accelerometer and gyroscope data by I2C protocol at a sampling rate of 100 Hz. The acceleration measurements were read in *g* units and transformed into units of *m*/*s*^2^ using *g* = 9.81 *m*/*s*^2^. The gyroscope measurements are in units of °/*s*. The MCU board was connected to the WiFi during its initialization. Then the inertial measurements were read from the MPU-9250, and sent using Open Sound Control (OSC) packages over UDP, following the defined rate. The smartphone and the MCU, screwed within a projected box, were attached to the leg using a band, as showed in Fig. 2. Five reflexive markers were taped in the band to calibrate it as a rigid body in the motion capture system, then at least three markers were kept during the walking trials to track it as a six-degree freedom object. These markers are also described in Table 1, at the fifth first lines. In the Fig. 3 is showed the motion capture software visualization of the coordinate reference calibrated for the optical motion capture system, smartphone and MCU attached to the leg being tracked as a rigid body. The MCU and smartphone were positioned to correspond to the motion capture reference in a way in which their coordinate systems are aligned.

An integration API was designed and developed to receive data from the MCU over UDP protocol and from the optical motion capture system by a real-time SDK provided by the Qualisys corporation^26^. The MCU’s inertial measurements and the 3D trajectories were acquired independently by this integration API but synchronously. The API starts to listen to the OSC packages from the MCU when the optical system begins the data acquisition. Also, the API stops listening to the OSC packages when the optical system finishes the data acquisition. Once the integration API guarantees both acquisitions initiate and finish together, and both systems have the same sampling rate, hence their data streams are synchronized.

### Data acquisition

Two data acquisition sessions were performed for each participant, and each one lasted about one hour. The sessions were performed on different days. In each session, the following procedure was adopted:

1. **Calibration of the systems**: the optical motion capture system was calibrated by the Wand calibration method following the manufacture’s instructions^24^. During the calibration, the coordinate system was defined as: *x* was the direction in which the participant walked; *y* was orthogonal to *x*; and *z* was orthogonal to both, pointing to the participants’ head. The MCU also was turned on at this moment, and its communication to the computer was verified. As well as, the Android application was started to read and store the accelerometer readings in a CSV file.
2. **Participant preparation**: the investigator showed the laboratory to the participant, explained the recording procedure, and asked the participant to sign the consent form. Further, the participant changed their clothes to tight-fitting outfits and wore tight caps to cover their hair. The investigator attached the reflexive markers presented in Fig. 1 on the participants’ skin or the tight-fitting clothes, using a proper double-sided tape. Also, the smartphone and the MCU box placed in the band were attached to the participant’s leg.
3. **Trials**: The participant was asked to stand up at the beginning of the walkway for few seconds to guarantee the proper function and communication of the systems. After these few seconds, the participant was asked to tap three times on the smartphone and MCU box (using their index finger, on which an additional marker was placed). Finally, the participant walked forth on a 5-m straight level walkway at their normal pace. At the end of the walkway, the participant stopped and tapped on the smartphone and MCU box again. The accelerations peaks generated by these taps allow a later verification of the data synchronization. Five trials of the participant walking using the band on their left leg were recorded, and five other sessions were recorded using the band placed on their right leg.
4. **Session ending**: After recording the ten trials, the band, and all the markers were removed from the participants’ bodies. The Android application also was stopped. The obtained visualizations from the session recordings were shown, and the scheduling of the participant’s next session was confirmed.

### Data processing

The marker trajectories were labeled using the QTM software (QTM version 2018.1 (build 4220), RT Protocol versions 1.0–1.18 supported), as well as their gap-filling procedure. The investigator manually selected the best fit of interpolation in the trajectory editor^27^ for each missing trajectory. Polynomial interpolation was applied in gaps smaller than ten frames, and relational interpolation, which is based on the movement of surrounding markers, was adopted for more complex cases (e.g., occlusions caused by the alignment of both legs during mid-stance and mid-swing phases). After that, the trajectories were smoothed, when necessary, using QTM software tool^27^ by selecting a range of the trajectories and employing the more appropriated filter. This filter was selected and applied using the QTM trajectory editor. Local spikes were identified using an acceleration threshold of 10 *m*/*s*^2^. Only these spikes were locally smoothed using the moving average filter of QTM trajectory editor. This approach was used to smooth local spikes without affecting the movements. The beginning and end of trials containing expressive high-frequency noise were smoothed using a Butterworth low pass filter with a 5 Hz cut-off frequency. This filter was applied only on the non-walking data, that corresponds to the first and final seconds of the trials in which the participants where preparing to start walking, as detailed in the Subsection **Data acquisition**. After that, these filled and smoothed trajectories were exported to the c3d (https://www.c3d.org) and Matlab file formats. These trajectories were then imported and processed using MoCap Toolbox^28^ under Matlab^29^. The exported trajectories were structured as *MoCap data* from MoCap Toolbox, and processed to extract the temporal section containing only walking data, i.e., removing the beginning and end of the trials. The second-order time derivatives (i.e. accelerations) from these walking data were estimated using the MoCap Toolbox, being also structured as *MoCap data*. The raw inertial measurements recorded by the MCU and smartphone were exported into CSV files, including the full trials, i.e., the beginning, walking data, and end of the trials. Additionally, the corresponding walking sections were extracted from these inertial data and exported to a CSV file. The walking sections obtained from the trajectories, MCU’s inertial measurements, and smartphone accelerations were assured to present temporal alignment.

## Data Records

All data are available from figshare^30^. They are organized in two folders: **raw_data** containing the complete trials; and **processed_data** storing the walking sections extracted from the raw data by removing the beginning and end of the trials. In both folders, the participants’ trial files are organized in sub folders associated to each participant identification: *userID*, which IDs are from 01 to 30, not necessarily consecutive. A total of 20 trials have been recorded of each participant, 10 in *day1* and 10 in *day2*.

### Raw data

This folder stores the complete trials, including the beginning and end of them. These trials consists in 3D trajectories exported from the QTM software into c3d files, and MCU’s inertial readings stored in CSV files. The c3d files are referenced as **capture_userID_dayD_TT_qtm.c3d**, and CSV files as **capture_userID_dayD_TT_imu.csv**, in which:

- **userID** is the participant identification, as explained before;
- **dayD** refers to the first or second day of the participant trials, **day1** or **day2**;
- **TT** is the trial number, i.e. 0001 to 0010, for each **dayD**.

The description of the c3d labels are presented in Table 1. All trajectories have the dimension n×3 (*n* is the number of frames recorded), and the number format is real. Information about the columns of CSV files are presented in Table 2. The corresponding frames to the inertial measurements are indicated in the first column of CSV files. In some cases, the frame counting of the MCU’s inertial measurements does not start in one, but they are surely synchronized to the marker trajectories’ frames.

**Table 2:** Description of the columns of the CSV files generated by the MCU’s inertial measurements during the participants’ trials.

Columns	Description	Format
**Frame**	Frame number to correspond to the marker trajectories' frames	Integer
**Acc x**	x-axis of accelerometer reading in the referenced frame number	Real (in units of *m*/*s*^2^)
**Acc y**	y-axis of accelerometer reading in the referenced frame number	Real (in units of *m*/*s*^2^)
**Acc z**	z-axis of accelerometer reading in the referenced frame number	Real (in units of *m*/*s*^2^)
**Gyro x**	x-axis of gyroscope reading in the referenced frame number	Real (in units of °/*s*)
**Gyro y**	y-axis of gyroscope reading in the referenced frame number	Real (in units of °/*s*)
**Gyro z**	z-axis of gyroscope reading in the referenced frame number	Real (in units of °/*s*)

### Processed data

This folder stores the walking sections obtained from the complete trials, removing the beginning and end of them. The walking sections of 3D trajectories were stored in Matlab files whereas the corresponding sections of inertial readings from the MCU and smartphone were stored in CSV files. The Matlab files are referenced as **capture_userID_dayD_TT_qtm_walk.mat**, and CSV files as **capture_userID_dayD_TT_imu_walk.csv** and **capture_userID_dayD_TT_nexus_walk.csv**, in which:

- **userID** is the participant identification, as explained before;
- **dayD** refers to the first or second day of the participant trials, **day1** or **day2**;
- **TT** is the trial number, i.e. 0001 to 0010, for each **dayD**.

First, the c3d files containing the markers trajectories were read over Matlab, and their data were stored in a structure array from MoCap Toolbox named *MoCap data*. The scheme and fields of this structure are described in Table 4. Format and set values of the fields are also presented. Some fields are composed by Structures, which are described on the columns to the right. Those fields kept as default values of *Mocap data* Structure are presented as empty or zero-valued in this table. Through the functions provided by Mocap Toolbox, walking sections were extracted by analysing the trajectories on *x*-axis of feet markers, medial and lateral malleolus of both sides (details in Table 1), to find the frame in which the participant displacement begins and ends. These frame numbers are stored in the field **frame_init** and **frame_end** of the Structure **other** of *Mocap data*.

Once the MCU’s inertial readings were synchronized to the trajectories by the corresponding frame numbers, these yielded beginning and ending frames were also used to section the MCU measurements. The columns and scheme of CSV files which store the walking sections extracted from MCU inertial data are the same of the presented in Table 1. The first value of column named **frame** presents the same amount of **frame_init**, and the last value presents the same amount of **frame_end** from the *MoCap data* Structure.

Aiming to facilitate comparison analyses between MCU’s inertial readings and markers’ data, second-order time derivatives (i.e., accelerations) were estimated from marker’s trajectories of walking sections. These data are also stored in *MoCap data* structures in Matlab files following the same nomenclature: **capture_userID_dayD_TT_qtm_acc_walk.mat**. The fields and values of this structure which stores the estimated accelerations are describe in Table 5. The schema, fields and format are basically the same of the trajectories’s *MoCap data* structure. However, the values in field **data** are the estimated accelerations of the corresponding trajectories (in units of *mm*/*s*^2^), and the value **2** of field **timederOrder** indicates that these data are the second-order time derivatives.

Accelerations from the smartphone were not capture synchronously to the marker trajectories, and only one CSV file stored all the accelerometer measurements of the 10 trials performed by a participant in each day. This CSV file was split into walking sections by correlation analysis between it and the walking sections of MCU accelerations, which were synchronized to the trajectories. Thus, these extracted walking sections from the smartphone accelerations do not contain the information about frame numbers although these were motion-aligned to the MCU’s accelerometer readings. The columns of the CSV files that store the walking sections of the smartphone accelerations are described in Table 3.

**Table 3:** Description of the columns of the CSV files generated by the smartphone accelerations during the participants’ trials.

Columns	Description	Format
Acc x	x-axis of the accelerometer measurement	Real (in units of *m*/*s*^2^)
Acc y	y-axis of the accelerometer measurement	Real (in units of *m*/*s*^2^)
Acc z	z-axis of the accelerometer measurement	Real (in units of *m*/*s*^2^)

**Table 4:** Description of the scheme, fields, their format and set values on *Mocap data* structure.

Field name	Format	Value			
**type**	String	‘MoCap data'			
**filename**	String				
**nFrames**	Integer	*f*			
**nCameras**	Integer	18			
**nMarkers**	Integer	47			
**freq**	Integer	100			
**nAnalog**	Integer	0			
**anaFreq**	Integer	0			
**timederOrder**	Integer	0			
**markerName**	String	47 × 1 String			
**data**	Real	*f* × 141 Real (in units of mm)			
**analogdata**	Real				
**other**	Structure	**descr**	String		
		**timeStamp**	String		
		**dataIncluded**	‘3D'		
		**RigidBodies**	Structure	**Bodies**	1
				**Name**	‘imu_nexus_box'
				**Positions**	1 × 3 × f Real (in units of mm)
				**Rotations**	1 × 9 × f Real (rotation matrices)
				**RPYs**	1 × 3 × f Real (in units of degrees–roll, pitch and yaw)
				**Residual**	1 × 1 × f Real (in units of mm)
		**frame_init**	Integer		
		**frame_end**	Integer		

*f is the number of frames of the walking section.

All fields’ names are in bold. The ones which format is Structure, their fields are detailed on the rightmost columns. The empty or zero-valued fields were not filled in the *Mocap data* structure.

**Table 5:** Description of the scheme, fields, their format and set values on *Mocap data* structure generated by the estimation of second-order time derivatives (i.e., accelerations) from the trajectories.

Field name	Format	Value			
**type**	String	‘MoCap data’			
**filename**	String				
**nFrames**	Integer	*f*			
**nCameras**	Integer	18			
**nMarkers**	Integer	47			
**freq**	Integer	100			
**nAnalog**	Integer	0			
**anaFreq**	Integer	0			
**timederOrder**	Integer	2			
**markerName**	String	47 × 1 String			
**data**	Real	*f* × 141 Real (in units of *mm*/*s*^2^)			
**analogdata**	Real				
**other**	Structure	**descr**	String		
		**timeStamp**	String		
		**dataIncluded**	‘3D'		
		**RigidBodies**	Structure	**Bodies**	1
				**Name**	‘imu_nexus_box'
				**Positions**	1 × 3 × f Real (in units of mm)
				**Rotations**	1 × 9 × f Real (rotation matrices)
				**RPYs**	1 × 3 × f Real (in units of degrees–roll, pitch and yaw)
				**Residual**	1 × 1 × f Real (in units of mm)
		**frame_init**	Integer		
		**frame_end**	Integer		

*f is the number of frames of the walking section.

All fields’ names are in bold. The ones which format is Structure, their fields are detailed on the rightmost columns. The empty or zero-valued fields were not filled in the *Mocap data* structure.

## Technical Validation

The presented multi-sensor dataset consists of three sources: an optical motion capture system, an IMU mounted to an ESP8266 board and an Android-based smartphone. The same experienced investigator attached the markers and the smartphone and MCU within a box mounted to a band on the participant’s body for consistency purposes.

### Optical 185 motion capture system

The calibration of the optical motion capture system was performed as described in the *Data acquisition* of the Section *Methods*, right before the beginning of each data acquisition session. In all achieved calibrations, the average residuals of each camera remained below 3 mm and similar among the 18 cameras. Also, the standard deviation between the actual wand length and the length perceived by the cameras was up to 1 mm in the adopted calibrations. The gaps in the 3D trajectories of the markers were filled (i.e., all the trajectories are 100% tracked), and the average residuals of 3D measured points fell below 4 mm.

### MCU

The firmware was implemented using Arduino Core libraries without adopting IMU libraries to avoid potentially applying of library default filters to the inertial measurements. Commonly, a scale range between ±2 *g* and ±8 *g* is adopted to the accelerometer in the gait literature^31^. The scale range of ±2 *g* is enough to measure the maximums instantaneous accelerations of normal walking (bellow to 1 *g*), and can capture more gait details (a scale range of ±2 *g* corresponds to 16384 *LSB*/*g* according to the MPU-9250 specification^25^). The scale range of ±250 was adopted to the gyroscope because it also presents the higher sensitivity scale factor (131 *LSB*/(°/*s*)). Thus, the firmware reads the registers’ position of the MPU-9250 accelerometer and gyroscope, then calculates the measurements according to each sensor’ sensitivity.

### Smartphone

The developed Android application uses the sensor routines provided by the platform^32^ and the Android Open Source Project (AOSP)^33^. The same scale range of ±2 *g* used to the MPU-9250 accelerometer was set to the MPU-6515 one using the Android Sensor API. This scale also corresponds to a specificity of 16384 *LSB*/*g* for the MPU-6515. The Android API reads these accelerations from the IMU and calculates them in units of *m*/*s*^2^, including the gravity.

### Data synchronization

We performed a quantification of the latency communication stages of the adopted setup consisting of Qualisys adopted optical motion capture system and a micro-controlled device communicating over UDP, in the same capture laboratory. We conducted event-to-end tests on the critical components of this setup to determine the synchronization suitability. This investigation showed suitability in the synchronization because the near individual average latencies of around 25 ms for both systems^34^.

## Usage Notes

The c3d files can be read by several free and open source tools which provide support to c3d files, such as EZC3D^35^ and Motion Kinematic & Kinetic Analyzer (MOKKA)^36^. The Matlab files store the structure *MoCap data* from MoCap Toolbox^28^, and can be manipulated using the functions provided by this Toolbox. It presents several routines to visualize, perform kinematics and kinetics analysis and apply projections on the data. This Toolbox supports any kind of marker set. The CSV format files can be read using any text or spreadsheet editor, as well as by common functions over Matlab (https://www.mathworks.com/help/matlab/ref/readtimetable.html) or Python (https://pandas.pydata.org/pandas-docs/stable/reference/api/pandas.read_csv.html).

## Data Availability

The developed Matlab and Python codes to process the data are freely available on the first author’s github repository (https://github.com/geisekss/motion_capture_analysis). The MoCap Toolbox is freely available and extensively documented on the University of Jyväskylä website (https://www.jyu.fi/hytk/fi/laitokset/mutku/en/research/materials/mocaptoolbox).
